# LSVT LOUD and LSVT BIG: Behavioral Treatment Programs for Speech and Body Movement in Parkinson Disease

**DOI:** 10.1155/2012/391946

**Published:** 2012-03-15

**Authors:** Cynthia Fox, Georg Ebersbach, Lorraine Ramig, Shimon Sapir

**Affiliations:** ^1^National Center for Voice and Speech, University of Colorado Boulder, Campus Box 409, Boulder, CO 80305, USA; ^2^Movement Disorders Clinic, Paracelsusring 6a, 14547 Beelitz-Heilstätten, Germany; ^3^Departments of Physiotherapy and Communication Sciences and Disorders, University of Haifa, Mount Carmel, Haifa 31905, Israel

## Abstract

Recent advances in neuroscience have suggested that exercise-based behavioral treatments may improve function and possibly slow progression of motor symptoms in individuals with Parkinson disease (PD). The LSVT (Lee Silverman Voice Treatment) Programs for individuals with PD have been developed and researched over the past 20 years beginning with a focus on the speech motor system (LSVT LOUD) and more recently have been extended to address limb motor systems (LSVT BIG). The unique aspects of the LSVT Programs include the combination of (a) an exclusive target on increasing amplitude (loudness in the speech motor system; bigger movements in the limb motor system), (b) a focus on sensory recalibration to help patients recognize that movements with increased amplitude are within normal limits, even if they feel “too loud” or “too big,” and (c) training self-cueing and attention to action to facilitate long-term maintenance of treatment outcomes. In addition, the intensive mode of delivery is consistent with principles that drive activity-dependent neuroplasticity and motor learning. The purpose of this paper is to provide an integrative discussion of the LSVT Programs including the rationale for their fundamentals, a summary of efficacy data, and a discussion of limitations and future directions for research.

## 1. Introduction

Progressive neurological diseases, such as Parkinson disease (PD) impair speech, swallowing, limb function, gait, balance, and activities of daily living. Even with optimal medical management (pharmacological, surgical) these deficits cannot be controlled satisfactorily in the vast majority of individuals with PD and have a negative impact on quality of life [1–3]. Recently, basic science research in animal models of PD has documented the value of exercise for improving motor performance and potentially slowing progression of motor symptoms and neural degeneration [4–9]. The impact of exercise in humans with PD is being increasingly explored in studies that incorporate key principles that have been identified to drive activity-dependent neuroplasticity (i.e., modifications in the central nervous system in response to physical activity), such as specificity, intensity, repetition, and saliency [9–16]. Collectively, these findings have accentuated the important role of exercise and/or rehabilitation in the overall management of PD. Previously, rehabilitation programs were often administered in later stages of PD or as reactive referrals for secondary impairments, such as aspiration due to swallowing dysfunction, or hip fracture due to falling. Today, such programs are being viewed as therapeutic options to be prescribed early in the course of PD that may potentially contribute to slowing of motor symptom progression [5, 17]. The purpose of this paper is to provide an integrative discussion of the rationale for and the efficacy of one type of rehabilitation approach, the LSVT Programs for speech (LSVT LOUD) and limb (LSVT BIG) motor systems in individuals with PD. We will include the rationale for targeting increased amplitude, the intensive mode of treatment delivery, and recalibration of the sensorimotor system including self-cueing, and attention to action, which may be important for generalization and long-term maintenance of treatment effects. In addition, we will summarize published efficacy data and discuss current limitations and future directions for research.

## 2. What Is LSVT LOUD?

Nearly 90% of individuals with PD have speech and voice disorders that negatively impact communication abilities [18, 19]. These disorders include reduced vocal loudness, monotone, hoarse, breathy voice quality, and imprecise articulation, perceived as mumbling, and other rate-related features, such as hesitations and short rushes of speech [20, 21]. In contrast to previous medical “chart review” literature suggesting a mid- or late-stage onset of speech and swallowing symptoms in PD [22], more recent investigations with sensitive and valid measures consistently report speech symptoms in early PD (e.g., [23]). Further, self-report data from individuals with PD have indicated that voice and speech changes are associated with inactivity, embarrassment, and withdrawal from social situations [2].

 Historically, speech treatment for individuals with PD was viewed as futile, in as much as treatment gains were minimal and short lived [24]. Today, LSVT LOUD is a standardized, research-based speech treatment protocol with established efficacy for PD [25–28]. LSVT LOUD trains the target of vocal loudness in order to (1) enhance the voice source, consistent with improving the carrier in the classic engineering concept of signal transmission [29], (2) use vocal loudness as a trigger for distributed effects (e.g., improved articulation, vocal quality and intonation, and reduced rate) across the speech production system [21, 30–33], (3) recalibrate sensorimotor perception of improved vocal loudness [34], and (4) train a single self-cue and attention to action to facilitate generalization of treatment effects into functional communication. Although LSVT LOUD is a standardized treatment protocol, the materials used during treatment and the homework and carryover exercises are made salient and tailored to each individual to facilitate motivation, engagement and the potential to drive neuroplasticity [13, 35, 36].

In contrast, traditional speech therapy typically involves multiple speech system targets (e.g., respiration, voice, articulation, and rate), is low intensity (1–2 sessions per week, minimal number of repetitions of treatment tasks), and does not systematically address the sensory processing deficits related to self-perception of loudness by individuals with PD (see [37] for summary table contrasting LSVT LOUD and traditional speech treatment) [37, 38]. The LSVT LOUD protocol is summarized in Table 1.

## 3. LSVT LOUD Outcome Data

Two randomized controlled trial (RCT) studies have been conducted [27, 28]. Data have documented that training increased vocal loudness results in a statistically significant and lasting increase in vocal sound pressure level (SPL) and frequency variability during speech (i.e., uncued conversational speech) as compared to a matched treatment focusing on training increased respiratory support [26–28, 30]. Effect size data for the primary outcome variable of vocal SPL in conversational speech were highly significant immediately posttreatment (1.20) and were maintained at 24 months posttreatment (1.03) [27, 30, 40]. Data providing initial external validation of LSVT LOUD outcomes have been reported by independent labs and reviews [41–45].

In addition, various physiologic changes such as increased movement amplitude of the rib cage (larger excursions) during speech breathing [46], increased subglottal air pressure [26], and improved closure and larger/more symmetrical movements of the vocal folds [47] have been documented in individuals with PD immediately after LSVT LOUD. These findings are supported by perceptual data demonstrating listeners rated improved loudness and voice quality in individuals with PD immediately posttreatment [33]. Subjects in these studies were predominately Hoehn and Yahr stages 1–3 with moderate speech deficits.

Training vocal loudness also has been studied for its distributed effects across the speech production system. In a series of smaller pilot studies (subsets of data from larger study) data have documented improvements in orofacial movements, as reflected in consonant articulation [48], tongue strength and motility [44], speech rate [30], ratings of improved facial expression [49], and improvements in some aspects of the oral phase of swallowing (e.g., reduced oral transit time) [50] even though these functions were not specific targets in therapy. The impact of LSVT LOUD on speech articulation, especially vowels, has been further explored. Vowels are formed and differentiated from each other by the movements of the tongue, lips, and jaw. In individuals with PD, these movements tend to be hypokinetic [51], thus rendering the vowels less distinct physiologically, acoustically, and perceptually, a phenomenon known as vowel centralization. LSVT LOUD has been shown to reduce vowel centralization and improve perceptual rating of vowel quality [31, 32]. This improvement may reflect larger amplitude of movements of the tongue, lips, and jaw, possibly due to overall neural and biomechanical coupling of speech subsystems and increased activation of the entire speech neuromuscular system [52].

Two brain imaging studies using O^15^ PET in a small number of individuals with PD have documented changes in brain function immediately following LSVT LOUD [53–55]. The most recent study by Narayana et al. [55] examined the neural mechanisms underlying the effects of training increased vocal loudness in ten individuals with PD and hypophonia. Cerebral blood flow during rest and reading conditions was measured by H_2_^15^O-positron emission tomography. *Z*-score images were generated by contrasting reading with rest conditions for pre- and post-LSVT LOUD sessions, and neural activity was correlated with the corresponding change in vocal SPL (loudness). Narayana et al. [55] hypothesized that brain activation patterns associated with LSVT LOUD training would reflect improved loudness, improved perception of self-generated voice output, and improved attention to action. Further it was hypothesized that these outcomes would likely be mediated via the right hemisphere and involve speech motor and premotor cortical areas (related to increasing vocal loudness), the auditory cortices (related to recalibration of perception of self-produced loudness), and dorsolateral prefrontal cortex (related to improving attention to action). To a large extent, the results of the study are consistent with these hypotheses. These initial neural findings underlying LSVT LOUD outcomes are being further examined and verified in ongoing imaging studies as discussed in ongoing research.

## 4. What Is LSVT BIG?

Individuals with PD perform movements that are hesitant (akinesia), slow (bradykinesia), and with reduced amplitude (hypokinesia). Changing from one motor program to another (set-shifting) may be disturbed and sequencing of repetitive movements may occur with prolonged and/or irregular intervals and reduced and/or irregular amplitudes [56]. External cues may exert disproportionate influences on motor performance and can trigger both motor blocks and kinesia paradoxica [57]. In LSVT BIG, training of amplitude rather than speed was chosen as the main focus of treatment to overcome bradykinesia/hypokinesia because training of velocity can induce faster movements but does not consistently improve movement amplitude and accuracy. Furthermore, training to increase velocity of limb movements may result in hypokinetic (reduced) movement amplitude [58, 59]. In contrast, training of amplitude not only results in bigger, but also in faster and more precise movement [58, 59]. The goal of LSVT BIG is to overcome deficient speed-amplitude regulation leading to underscaling of movement amplitude at any given velocity [59–61]. Continuous feedback on motor performance and training of movement perception is used to counteract reduced gain in motor activities resulting from disturbed sensorimotor processing [62].

 Most current therapies rely on compensatory behavior and external cueing in order to bypass deficient basal ganglia function [58, 63–70]. In contrast, other protocols focus on retraining of deficient functions. Task-specific, repetitive, high-intensity exercises for individuals with PD include treadmill training [71], training of compensatory steps [72] walking [73], and muscle strengthening [74, 75]. LSVT BIG belongs to the latter restorative approaches and is aiming to restore normal movement amplitude by recalibrating the patient's perception of movement execution. LSVT BIG differs from other forms of physiotherapy in PD in its training of movement amplitude as a single treatment parameter (both single motor target and cognitive cue) through high effort, intensive treatment with a focus on recalibrating sensory perception of normal amplitude of movements. The standardized protocol of LSVT BIG was derived directly from LSVT LOUD and is summarized in Table 1.

## 5. LSVT BIG Outcome Data

Presently two trials on the effectiveness of LSVT BIG have been published.

A noncontrolled study assessed effects of LSVT BIG in 18 individuals with PD [76]. Data documented that after four weeks of training, subjects demonstrated a modest (12%–14%) increase in velocity of walking and reaching movements.

 In the recently published rater-blinded Berlin LSVT BIG Study improvement in motor performance was compared in 60 individuals with PD, randomly assigned to receive LSVT BIG, Nordic Walking (as group treatment) or domestic training without supervision [60]. Mean improvement of UPDRS motor score in subjects receiving LSVT BIG was 5.05 at four-month followup. In contrast, the UPDRS motor score slightly deteriorated in control groups undergoing training in Nordic walking with the same amount of supervised sessions and in subjects who received domestic training receiving a 1-hour instructional lesson and no further supervision by a therapist. The beneficial outcome in LSVT BIG was also reflected by improvements in further assessments including a standard time-up and go task and 10-meter walk. According to Schrag et al. [77] a change of five points is the most appropriate cutoff score for the minimal clinical important change (MCIC) of the UPDRS motor score for all Hoehn and Yahr stages from Stage I to III. The degree of change in UPDRS motor score after LSVT BIG can thus be assumed to be clinically relevant. There is no established definition of the MCIC for the secondary motor assessments, but the observed 10–15% improvements in timed test of mobility are likely to have functional impact.

 The Berlin LSVT BIG Study is one of the few studies comparing specific types of physiotherapy with both active comparators and inactive controls. Sage and Almeida [78] reported more improvement in the UPDRS motor score and other motor tasks with exercises designed to improve sensory attention and body awareness when compared to lower-limb aerobic training. Mak and Hui-Chan 2008 [79] found better outcomes in the Sit-and-Stand task when subjects received training including sensory as compared to conventional exercise. In both studies individuals without active interventions did not improve. In the Berlin LSVT BIG Study outcomes differed clearly between active interventions. Intensive one-to-one training (LSVT BIG) was found to be more effective than Nordic walking delivered as group training. Although differences in training techniques may also have influenced results, it is likely that the specific protocol of LSVT BIG and, possibly, individual face-to-face interaction between patient and therapist, was more crucial for successful outcomes than total exercise time. Further studies are needed to explore differences in cost-effectiveness between the more expensive individual LSVT BIG training, group treatments, and self-supervised domestic exercise.

## 6. Unique Fundamentals of LSVT Programs

### 6.1. Target: Amplitude

We hypothesize that training-induced increases in movement amplitude target the proposed pathophysiological mechanisms underlying bradykinesia/hypokinesia—inadequate muscle activation [62]. The muscle activation deficits that occur in bradykinesia are believed to result from inadequate merging of kinesthetic feedback, motor output, and context feedback within the basal ganglia, necessary to select and reinforce an appropriate gain in the motor command [62, 80]. Although the target is increased amplitude, it is important to note that the end result in speech and movement amplitude output (louder voice/bigger movements) is within normal limits. The cue of “loud” or “big” is used to simply drive increased motor output across the motor systems for more normal amplitude. The role of the speech, physical, or occupational therapist is to shape the amplitude into healthy, good quality movements (see Shaping in Table 1). Post-LSVT LOUD videostroboscopic data [47] and perceptual ratings of voice [33] indicate improved laryngeal function and voice quality rather than vocal hyperfunction or deterioration in voice posttreatment. Ratings of motor performance after LSVT BIG also indicated a trend towards normality and no exaggeration or overcompensation of movement amplitudes [60, 76, 81].

The idea of targeting amplitude in rehabilitation for individuals with PD is not new. Training vocal loudness (amplitude) is consistent with approaches recommended for treating motor speech disorders that (a) create a single motor organizing theme, (b) have a maximum impact on other aspects of speech production, and (c) increase effort across the speech mechanism [81–83]. Further, many physical therapy programs have amplitude as a component of therapy either as exercise principles or by using external cues (e.g., [84, 85]). The unique element of training amplitude in LSVT Programs is that it is the *exclusive* focus. We hypothesize that a single, overlearned cue (louder voice/bigger movements) may minimize cognitive load and mental effort [86] and possibly facilitate maintenance and generalization of treatment strategies outside of the therapy room. This hypothesis is yet to be formally tested and is an area for future research. For example, testing the impact of dual task functioning on the ability of individuals with PD to maintain improved amplitude before/after LSVT Programs would elucidate the ability of these individuals to learn a new self-cue for amplitude.

### 6.2. Mode: Intensive, High Effort Therapy

The training mode of LSVT Programs is consistent with some principles that promote activity-dependent neuroplasticity [11, 87] including (a) specificity, targeting bradykinesia/hypokinesia through increasing amplitude of motor output, (b) intensity, increased dosage of treatment, (c) repetition, increased repetition of tasks (minimum 15 repetitions) within treatment sessions and home practice, and (d) saliency of treatment tasks, individualized hierarchy and carryover assignments for active practice of desired goals, interests and abilities of each person [9–16]. Further, we recognize that acquisition of the motor skill (e.g., louder voice, bigger movements) alone may not be sufficient for sustained neuroplasticity (i.e., sensorimotor map reorganization, synaptogenesis) [14] or for carryover and long-term maintenance outside the therapeutic environment. Therefore, a direct translation of the structured motor exercises (daily exercises) into functional daily activities is emphasized in treatment with the goal of facilitating generalization outside of the treatment room (see Table 1 Hierarchy, Carryover and Homework). In addition, emphasis is placed on establishing life-long habits of structured homework practice of voice/movement exercises that continue beyond the one-month of treatment. Finally, simply using the louder voice or bigger movements in daily living provides additional practice, as summarized by this patient quote,

“in my normal everyday life, I just exaggerate my movements. I keep things big when I reach for things, or when I bend or when I walk; and when I talk–I keep my voice loud.”

### 6.3. Recalibration: Addressing Barriers to Generalization

Sensorimotor processing deficits during speech and movement have been well documented [37, 38, 88–91]. From our own clinical observations, it appears that addressing the motor deficit in isolation is not sufficient for lasting treatment outcomes that generalize beyond the treatment room [34]. Thus, the LSVT Programs are designed to train individuals with PD to recalibrate their motor and perceptual systems so that they are less inclined to downscale (reduce amplitude) speech and limb movement parameters after treatment.

**Figure 1:**
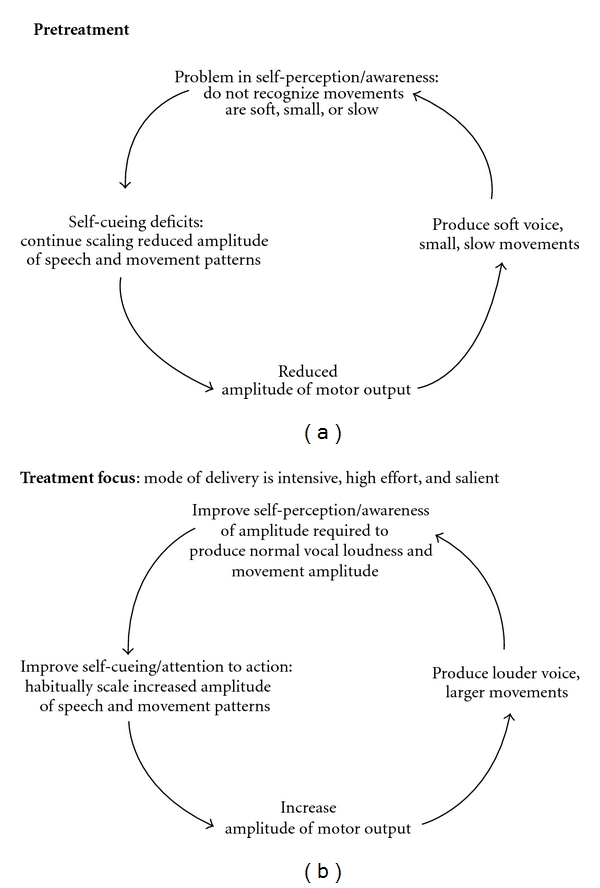
We hypothesize that pretreatment (a), individuals with PD have reduced amplitude of motor output, which results in soft voice and small movements. Due to problems in sensory self-perception they are not aware of the soft voice and small movements, or they do not recognize the extent of their soft voice and smaller movements. As a result, no error correction is made and individuals continue to program or self-cue reduced amplitude of motor output. They are “stuck” in a cycle of being soft and small. The focus in treatment (b) is on increasing the amplitude of motor output by having individuals with PD produce a louder voice and larger movements. Individuals are then taught that what feels/sounds/looks “too loud” or “too big” is within normal limits and has a positive impact on daily functional living. Therefore at the end of treatment, individuals habitually self-cue increased amplitude of motor output and have attention to action. Now they are in a cycle of a louder voice and bigger movements.

Figure 1 illustrates our hypothesized model for amplitude rescaling and recalibration in LSVT Programs. In short, the goal is to teach individuals with PD to produce motor output required for louder voice and bigger movements (Figure 1(b)) and help them recognize that this increased output results in within normal limits voice and movements. Directly addressing this sensory mismatch may help individuals learn to habitually (i.e., self-cue) speak with greater vocal loudness and move with bigger movements at the end of therapy. A specific example of a recalibration task includes recording the individual's voice while reading in a voice that they self-perceive as “too loud” and then playing it back to them. Individuals with PD can recognize when they hear the audio recordings that what felt and sounded too loud to them while reading, actually sounds within normal limits (or in some cases still too soft). Similarly, video recording an individual with PD as they walk or move in a manner that they perceive as “too big” allows them to visualize that what felt too big to them actually looks like normal movements (or in some cases still too small). Additional recalibration activities are detailed in Table 1.

The hypothesized concepts underlying recalibration in LSVT Programs have yet to be systematically tested in pre/posttreatment experiments. However, there is evidence that cognitive training is possible in individuals with PD [92], including training in motor attention to action and performance under multiple tasks [93, 94]. Moreover, the ability to speak in a louder voice two years after intervention as compared to pretreatment levels [27] support the ability of LSVT-LOUD-trained individuals to self-monitor vocal loudness at some level.

## 7. Limitations of LSVT Programs

There are a number of limitations to the scope of research on LSVT Programs and we have highlighted some of the key areas below. First, there is a need to better define prognostic variables for who will respond best to LSVT Programs and what outcomes can be expected in individuals with a variety of factors, such as depression, dementia, apathy, orthopedic complications, and dyskinesias, as well as atypical PD and post-DBS surgery. While the majority of LSVT outcome data have been reported on individuals with idiopathic PD, single subject, case study and small group designs have documented post-LSVT-LOUD improvements in individuals after neurosurgery and with atypical parkinsonism [95–97]. However, these outcomes may not be of the same magnitude as those observed in individuals with mild-to-moderate idiopathic PD and these individuals may require more frequent follow-up treatment sessions to maintain improvements over time. Furthermore, LSVT LOUD outcomes in individuals with significant rate disorders, such as palilalia, and individuals who have severe speech disorders secondary to high-frequency DBS stimulation have been poor. Data examining LSVT BIG in atypical and post-DBS populations are not available. Second, studies examining the optimal dose-response relationships for LSVT Programs across idiopathic PD, atypical PD, and individual post-DBS are needed. The standard dose of LSVT Programs is 16 individual 60-minute sessions within one month. There is one dose-response study for LSVT LOUD that examined the impact of an extended treatment protocol (LSVT Extended, LSVT-X) [98]. Specifically, individuals received in-person treatment two days a week and completed home practice sessions the other two days a week for 8 weeks of treatment. Outcome data immediately posttreatment were comparable to the standard dosage. Of note, the treating clinicians completed daily calls and extensive home-practice monitoring to ensure that all subjects completed all home sessions. Ongoing work is examining alternative dosages of LSVT BIG, additional dose-response relationships need to be defined. Third, the spread of effects across the speech production system has been reported following LSVT LOUD. These studies should be further advanced and studies are needed to evaluate the spread of effects or transfer effects from large body movements to fine motor functions, balance, or dual tasks following LSVT BIG. Fourth, the practical and financial feasibility of delivering intensive treatment in LSVT Programs must be addressed. Physical immobility and geographical constraints are barriers which limit patient accessibility to intensive treatment. Fifth, the maintenance and enhancement of long-term treatment effects also are areas of need. While LSVT LOUD outcome data report maintenance of treatment effects for two years after one month of treatment, we believe outcomes can be further optimized. The long-term effects of LSVT BIG need to be established. Strategies to maximize compliance with continued home practice and the timing of optimal follow-up treatment intervals need to be defined. Finally, the hypothesized concepts underlying sensory calibration as well as understanding neural mechanisms of treatment-related change need to be systematically studied and validated. Only then can we fully understand what elements of treatment contribute to improvement in speech and movement functioning. These limitations will continue to guide our future research with some areas already being addressed as discussed below.

## 8. Current and Future Research Directions

Our ongoing work in LSVT LOUD is addressing questions related to the importance of the treatment target versus the mode of delivery. Specifically, we are comparing two treatment targets: vocal loudness training (LSVT LOUD) versus orofacial/articulation training (LSVT ARTIC) and the effects on measures of speech intelligibility, speech acoustics, facial expression, and swallowing. The two treatments are standardized and matched in terms of mode of delivery (e.g., dosage, sensory recalibration, homework, and carryover assignments). LSVT LOUD focuses on training healthy vocal loudness across speech tasks (sustained vowels, high/low vowels, functional phrases, and speech hierarchy), with focused attention on how it feels and sounds to talk LOUD, whereas, LSVT ARTIC focuses on high-force articulation or enunciation across speech tasks (diadochokinesis, contrastive pairs, functional phrases, and speech hierarchy), with focus on how it feels to have high-effort enunciation. Preliminary data examining single-word intelligibility in noise conditions [99] and facial expressions utilizing the Facial Action Coding System (FACS) [100] revealed significant improvements from pre to posttreatment in the LSVT LOUD group only. More extensive analysis is ongoing. In addition, this study includes comprehensive neuropsychological profiles of subjects and may shed some light on the impact of factors such as age, stage of disease, depression, dementia, or other nonmotor symptoms on treatment outcomes.

 The impact of DBS on speech is an urgent area of research. Tripoliti and colleagues [101] are assessing the reasons for the heterogeneous speech outcomes following DBS-STN by involving simultaneous quantitative measures of pre- and postsurgical speech functioning and details of surgical and stimulator optimization. Knowledge gained from these studies is likely to facilitate development of treatment approaches for speech problems in individuals with DBS-STN either before surgery (as preventative) or after surgery (as rehabilitation). Our laboratory is looking at the impact of additional weeks of treatment on speech outcomes for individuals with PD after DBS.

Advances in computer and web-based technology offer potentially powerful solutions to the problems of treatment accessibility, efficacious dosage delivery, and long-term maintenance in rehabilitation [102–104]. Preliminary studies have documented the impact of telepractice and software programs on treatment availability for LSVT LOUD and suggest that such technology may be effective [42, 105–108] and increase the feasibility of intensive dosage and long-term followup. In addition, a study by Tindall et al. [105] completed a cost analysis comparing in-person delivery of LSVT LOUD versus telepractice delivery. The computed mean amount of time and money for individuals with PD across these two modes of delivery was reported. The live delivery mode required 51 hours for 16 visits (travel and therapy time), $953.00 on fuel/mileage expenses, and $269.00 for other expenses (e.g., food). In contrast, the telepractice delivery option required 16 hours of time (therapy, no travel) and no additional costs for fuel/mileage or other expenses. To further enhance accessibility, a software program designed to collect acoustic data and provide interactive feedback as it guides the patient through the LSVT LOUD exercises has been developed. Outcome data document that treatment effects are comparable when half of the sessions were delivered by software [109]. These studies need further validation. While telepractice has not been explored for delivery of LSVT BIG, there are studies that have documented the feasibility of remote measuring of activities of daily living [110] and ongoing trials examining the delivery of physical therapy via telepractice in patients after stroke [111]. Thus, future applications of both telepractice and software programs/gaming technology to increase accessibility and feasibility of LSVT BIG is possible. The use of technology is not LSVT specific and may have the ability to increase accessibility, enhance effectiveness, and reduce financial burden of many intensive rehabilitation programs for people with PD.

 Understanding neural mechanisms of both speech and movement disorders in PD as well as mechanism of treatment-related change are of great promise to help improve treatment outcomes. As part of our ongoing work we are examining neural changes (PET imaging) in individuals with PD across the LSVT LOUD, LSVT ARTIC, and Untreated groups. Hypothetically, intensive practice of speech enunciation by the LSVT ARTIC regimen should strengthen cortically mediated speech articulation in PD, beyond the improvement associated with LSVT LOUD. To our knowledge, this will be the first imaging study of comparison speech treatments in individuals with PD including long-term followup (3 months). Developing parallel imaging studies before/after LSVT BIG is of great interest to us both in terms of understanding reorganization of brain activation patterns following treatment but also to understand differences between using amplitude to treat speech versus limb motor systems.

 Finally, whereas studies of movement and limb/gait exercise in animal models of PD have contributed immensely to the literature, there have been no analogous models for studying vocalization deficits. Today, emerging models of vocal motor deficits following dopamine depletion in rodents (rats and mice) and songbirds offer promise for the feasibility and value of these models [112–114]. Viable animal models of vocalization patterns associated with PD may allow us to accelerate the acquisition of the neurobiological and behavioral evidence to improve our understanding of voice/speech deficits in PD and document the therapeutic value of early interventions to slow voice/speech symptom progression in human PD.

 Collectively these ongoing studies have the potential to improve our understanding of the underlying mechanisms of speech-treatment-related changes in individuals with PD and will help guide treatment improvements. Future research will address the underlying bases for treatment-related changes that have a beneficial impact on speech and movement and thus quality of life in individuals with Parkinson disease.

## Figures and Tables

**Table 1:** Comparison of LSVT LOUD and LSVT BIG treatments.

*LSVT LOUD *(e.g., [25, 30])	*LSVT BIG *(e.g., [39])
*Target: LOUD*	*Target: BIG*

Increased movement amplitude directed predominately to respiratory/laryngeal systems	Increased movement amplitude directed across limb motor system including gait

*Intensity*: standardized	*Intensity*: standardized

Dosage: 4 consecutive days a week for 4 weeks (16 sessions in one month) Repetitions: minimum 15 repetitions/task Effort: push for maximum patient-perceived effort each day (8 or 9 on scale of 1–10 with 10 being the most)	Dosage: 4 consecutive days a week for 4 weeks (16 sessions in one month) Repetitions: minimum 8–16 repetitions/task Effort: push for maximum patient-perceived effort each day (8 or 9 on scale of 1–10 with 10 being the most)

*Daily exercises *	*Daily exercises*

First half of the treatment session (30 min.) * Task 1: Maximum Sustained Movements* 15 reps: sustain “ah” in Loud good quality voice as long as possible * Task 2: Directional Movements * 15 reps each: say “ah” in Loud good quality voice going high in pitch; 15 reps each: say “ah” in Loud good quality voice going low in pitch * Task 3: Functional Phrases * Patient self-identifies 10 phrases or sentences he/she says daily in functional living (e.g., “Good morning”) 5 reps of the list of 10 phrases. “Read phrases using same effort/loudness as you did during the long “ah”	First half of the treatment session (30 min. or more) * Task 1: Maximum Sustained Movements: seated * 8 reps: sustain Big “stretch” floor to ceiling (10 sec hold); 8 reps: sustain Big “stretch” side to side (10 sec hold) * Task 2: Repetitive/Directional Movements: standing * 16 reps: Forward Big step – 8 each leg; 16 reps: Sideways Big step – 8 each side; 16 reps: Backward Big step – 8 each leg; 20 reps: Forward Big Rock and reach – 10 each side; 20 reps: Sideways Big Rock and reach – 10 each side *Task 3: Functional Component Movements * Patient self-identifies 5 movements he/she does in functional living every day (e.g., Sit-to-stand) Clinician and patient select one simple component of each of these movements 5 reps each of the 5 component movements “Do your movement with the same effort/bigness that you did during the daily exercises”

*Hierarchy *	*Hierarchy*

Second half of the treatment session (30 min) (i) Designed to train rescaled amplitude/effort of movement achieved in daily exercises and functional phrases into in context specific and variable speaking activities (ii) Tasks increase complexity across weeks (Words-phrases-sentences-reading-conversation) and can be tailored to each patient's goals and interests (e.g., golf versus cooking) (iii) Tasks progress in difficulty by increasing duration (maintain LOUD for longer periods of time) amplitude (loudness, within normal limits), and complexity of tasks (dual processing, background noise, and attentional distracters)	Second half of the treatment session (30 min or less) (i) Designed to train rescaled amplitude/effort of movement achieved in daily exercises and functional component movements into in context specific and variable movement activities (ii) Complex multilevel tasks that progressively become more difficult over the 4 weeks and can be tailored to each patient's goals and interests (e.g., basic bathroom skills versus going out to dinner or shopping) (iii) Tasks progress in difficulty by increasing duration (maintain BIG for longer periods of time) amplitude (bigness/effort, within normal limits), and complexity of tasks (multisteps, dual processing, background noise, and attentional distracters) (iv) BIG walking is included as part of hierarchy on a daily basis. Time and distance will vary across patients, hierarchy goals, and weeks of therapy

*Shaping techniques*	*Shaping techniques*

Goal: train vocal loudness that is healthy and good quality (i.e., no unwanted vocal strain or excessive vocal fold closure) Technique: shape the quality and voice loudness through use of modeling or tactile/visual cues. “Watch me and do what I do.” Minimal cognitive loading: behavior is not achieved through extensive instructions or explanations, which are often too complex for patient to generalize outside of treatment room, but rather the patient is trained through modeling	Goal: train movement bigness that is healthy and good quality (i.e., no unwanted strain or pain, impingement, or awkward biomechanics) Technique: shape the quality and movement bigness through use of modeling or tactile/visual cues. “Watch me and do what I do.” Minimal cognitive loading: behavior is not achieved through extensive instructions or explanations, which are often too complex for patient to generalize outside of treatment room, but rather the patient is trained through modeling

*Sensory recalibration*	*Sensory recalibration*

Treatment: focus attention on how it feels and sounds to talk LOUD Carryover activities: start day one; daily assignments (treatment and nontreatment days); Loud good quality voice in real-life situations; (i) difficulty of the assignment matches the level of the hierarchy where the person is working; (ii) make patient accountable and probe for comments from patient that people in their daily living have said, such as, “I can hear you better” Homework practice: start day one: daily assignments to practice at home (Daily Exercises and Hierarchy Exercises); treatment days (one other time for 5–10 minutes); nontreatment days (two times for 10–15 minutes); homework book provided and patient made accountable	Treatment: focus attention on how it feels and looks to move BIG Carryover activities: start day one; daily assignments (treatment and nontreatment days); use big movements in real-life situations; (i) difficulty of the assignment matches the level of the hierarchy where the person is working; (ii) make patient accountable and probe for comments from patient that people in their daily living have said, such as, “You are moving better” Homework practice: start day one: daily assignments to practice at home (Daily Exercises and Functional Component Movements, Walking BIG); treatment days (one other time for 5–10 minutes); nontreatment days (two times for 10–15 minutes); homework book provided and patient made accountable

## References

[1] Keus SHJ, Munneke M, Nijkrake MJ, Kwakkel G, Bloem BR (2009). Physical therapy in Parkinson’s disease: evolution and future challenges. *Movement Disorders*.

[2] Miller N, Noble E, Jones D, Burn D (2006). Life with communication changes in Parkinson’s disease. *Age and Ageing*.

[3] Ransmayr G (2011). Physical, occupational, speech and swallowing therapies and physical exercise in Parkinson's disease. *Journal of Neural Transmission*.

[4] Fisher BE, Wu AD, Salem GJ (2008). The effect of exercise training in improving motor performance and corticomotor excitability in people with early Parkinson’s disease. *Archives of Physical Medicine and Rehabilitation*.

[5] Hirsch MA, Farley BG (2009). Exercise and neuroplasticity in persons living with Parkinson’s disease. *European Journal of Physical and Rehabilitation Medicine*.

[6] Petzinger GM, Fisher BE, Van Leeuwen JE (2010). Enhancing neuroplasticity in the basal ganglia: the role of exercise in Parkinson’s disease. *Movement Disorders*.

[7] Tillerson JL, Miller GW (2002). Forced limb-use and recovery following brain injury. *Neuroscientist*.

[8] Tillerson JL, Caudle WM, Reverón ME, Miller GW (2003). Exercise induces behavioral recovery and attenuates neurochemical deficits in rodent models of Parkinson’s disease. *Neuroscience*.

[9] Tillerson JL, Cohen AD, Philhower J, Miller GW, Zigmond MJ, Schallert T (2001). Forced limb-use effects on the behavioral and neurochemical effects of 6-hydroxydopamine. *Journal of Neuroscience*.

[10] Kleim JA, Jones TA (2008). Principles of experience-dependent neural plasticity: implications for rehabilitation after brain damage. *Journal of Speech, Language, and Hearing Research*.

[11] Kleim JA, Jones TA, Schallert T (2003). Motor enrichment and the induction of plasticity before or after brain injury. *Neurochemical Research*.

[12] Vučcković MG, Li Q, Fisher B (2010). Exercise elevates dopamine D2 receptor in a mouse model of Parkinson’s disease: in vivo imaging with [18F]fallypride. *Movement Disorders*.

[13] Conner JM, Chiba AA, Tuszynski MH (2005). The basal forebrain cholinergic system is essential for cortical plasticity and functional recovery following brain injury. *Neuron*.

[14] Kleim JA, Hogg TM, VandenBerg PM, Cooper NR, Bruneau R, Remple M (2004). Cortical synaptogenesis and motor map reorganization occur during late, but not early, phase of motor skill learning. *Journal of Neuroscience*.

[15] Kleim JA, Cooper NR, VandenBerg PM (2002). Exercise induces angiogenesis but does not alter movement representations within rat motor cortex. *Brain Research*.

[16] Remple MS, Bruneau RM, VandenBerg PM, Goertzen C, Kleim JA (2001). Sensitivity of cortical movement representations to motor experience: evidence that skill learning but not strength training induces cortical reorganization. *Behavioural Brain Research*.

[17] Zigmond MJ, Cameron JL, Leak RK (2009). Triggering endogenous neuroprotective processes through exercise in models of dopamine deficiency. *Parkinsonism and Related Disorders*.

[18] Ho AK, Iansek R, Marigliani C, Bradshaw JL, Gates S (1998). Speech impairment in a large sample of patients with Parkinson’s disease. *Behavioural Neurology*.

[19] Logemann JA, Fisher HB, Boshes B, Blonsky ER (1978). Frequency and cooccurrence of vocal tract dysfunctions in the speech of a large sample of Parkinson patients. *Journal of Speech and Hearing Disorders*.

[20] Darley FL, Aronson AE, Brown JR (1969). Clusters of deviant speech dimensions in the dysarthrias. *Journal of Speech and Hearing Research*.

[21] Sapir S, Pawlas AA, Ramig LO (2001). Voice and speech abnormalities in Parkinson disease: relation to severity of motor impairment, duration of disease, medication, depression, gender, and age. *Journal of Medical Speech-Language Pathology*.

[22] Müller J, Wenning GK, Verny M (2001). Progression of dysarthria and dysphagia in postmortem-confirmed parkinsonian disorders. *Archives of Neurology*.

[23] Rusz J, Cmejla R, Ruzickova H, Ruzicka E (2011). Quantitative acoustic measurements for characterization of speech and voice disorders in early untreated Parkinson's disease. *Journal of the Acoustical Society of America*.

[24] Sarno MT (1968). Speech impairment in Parkinson’s disease. *Archives of Physical Medicine and Rehabilitation*.

[25] Ramig L, Pawlas A, Countryman S (1995). *Lee Silverman Voice Treatment: A Practical Guide to Treating the Voice and Speech Disorders in Parkinson Disease*.

[26] Ramig LO, Dromey C (1996). Aerodynamic mechanisms underlying treatment-related changes in vocal intensity in patients with Parkinson disease. *Journal of Speech, Language, and Hearing Research*.

[27] Ramig LO, Sapir S, Countryman S (2001). Intensive voice treatment (LSVT®) for individuals with Parkinson’s disease: A two year follow-up. *Journal of Neurology Neurosurgery and Psychiatry*.

[28] Ramig LO, Sapir S, Fox C, Countryman S (2001). Changes in vocal loudness following intensive voice treatment (LSVT®) in individuals with Parkinson’s disease: a comparison with untreated patients and nornal age-matched controls. *Movement Disorders*.

[29] Titze I (1993). *Vocal Fold Physiology: Frontiers in Basic Science*.

[30] Ramig LO, Countryman S, Thompson LL, Horii Y (1995). Comparison of two forms of intensive speech treatment for Parkinson disease. *Journal of Speech and Hearing Research*.

[31] Sapir S, Spielman JL, Ramig LO, Story BH, Fox C (2007). Effects of intensive voice treatment (the Lee Silverman Voice Treatment [LSVT]) on vowel articulation in dysarthric individuals with idiopathic Parkinson disease: acoustic and perceptual findings. *Journal of Speech, Language, and Hearing Research*.

[32] Sapir S, Ramig LO, Spielman JL, Fox C (2010). Formant centralization ratio: a proposal for a new acoustic measure of dysarthric speech. *Journal of Speech, Language, and Hearing Research*.

[33] Baumgartner CA, Sapir S, Ramig LO (2001). Voice quality changes following phonatory-respiratory effort treatment (LSVT®) versus respiratory effort treatment for individuals with Parkinson disease. *Journal of Voice*.

[34] Fox CM, Morrison CE, Ramig LO, Sapir S (2002). Current perspectives on the Lee Silverman voice treatment (LSVT®) for individuals with idiopathic Parkinson disease. *American Journal of Speech-Language Pathology*.

[35] Alexander GE, Crutcher MD (1990). Functional architecture of basal ganglia circuits: neural substrates of parallel processing. *Trends in Neurosciences*.

[36] Graybiel AM (2005). The basal ganglia: learning new tricks and loving it. *Current Opinion in Neurobiology*.

[37] Ho AK, Bradshaw JL, Iansek R (2000). Volume perception in Parkinsonian speech. *Movement Disorders*.

[38] Ho AK, Bradshaw JL, Iansek R, Alfredson R (1999). Speech volume regulation in Parkinson’s disease: effects of implicit cues and explicit instructions. *Neuropsychologia*.

[39] Farley BG, Fox CM, Ramig LO, McFarland DH (2008). Intensive amplitude-specific therapeutic approaches for Parkinson’s disease: toward a neuroplasticity-principled rehabilitation model. *Topics in Geriatric Rehabilitation*.

[40] Sapir S, Ramig LO, Fox CM (2011). Intensive voice treatment in Parkinson's disease: Lee Silverman voice treatment. *Expert Review of Neurotherapeutics*.

[41] Constantinescu G, Theodoros D, Russell T, Ward E, Wilson S, Wootton R (2011). Treating disordered speech and voice in Parkinson's disease online: a randomized controlled non-inferiority trial. *International Journal of Language and Communication Disorders*.

[42] Howell S, Tripoliti E, Pring T (2009). Delivering the Lee Silverman Voice Treatment (LSVT) by web camera a feasibility study. *International Journal of Language and Communication Disorders*.

[43] Theodoros DG, Thompson-Ward EC, Murdoch BE, Lethlean J, Silburn P (1999). The effects of the Lee Silverman Voice Treatment Program on motor speech function in Parkinson disease following thalamotomy and pallidotomy surgery: a case study. *Journal of Medical Speech-Language Pathology*.

[44] Ward EC, Theodoros DG, Murdoch BE, Silburn P (2000). Changes in maximum capacity tongue function following the Lee Silverman Voice Treatment Program. *Journal of Medical Speech-Language Pathology*.

[45] Yorkston KM, Hakel M, Beukelman DR, Fager S (2007). Evidence for effectiveness of treatment of loudness, rate, or prosody in dysarthria: a systematic review. *Journal of Medical Speech-Language Pathology*.

[46] Huber JE, Stathopoulos ET, Ramig LO, Lancaster SL (2003). Respiratory function and variability in individuals with parkinson disease: pre- and post-Lee Silverman voice treatment. *Journal of Medical Speech-Language Pathology*.

[47] Smith ME, Ramig LO, Dromey C, Perez KS, Samandari R (1995). Intensive voice treatment in Parkinson disease: laryngostroboscopic findings. *Journal of Voice*.

[48] Dromey C, Ramig LO, Johnson AB (1995). Phonatory and articulatory changes associated with increased vocal intensity in Parkinson disease: a case study. *Journal of Speech and Hearing Research*.

[49] Spielman JL, Borod JC, Ramig LO (2003). The effects of intensive voice treatment on facial expressiveness in Parkinson disease: preliminary data. *Cognitive and Behavioral Neurology*.

[50] El Sharkawi A, Ramig L, Logemann JA (2002). Swallowing and voice effects of Lee Silverman Voice Treatment: a pilot study. *Journal of Neurology Neurosurgery and Psychiatry*.

[51] Forrest K, Weismer G, Turner GS (1989). Kinematic, acoustic, and perceptual analyses of connected speech produced by Parkinsonian and normal geriatric adults. *Journal of the Acoustical Society of America*.

[52] McClean MD, Tasko SM (2002). Association of orofacial with laryngeal and respiratory motor output during speech. *Experimental Brain Research*.

[53] Liotti M, Vogel D, Sapir S, Ramig L, New P, Fox P Abnormal auditory gating in Parkinson's disease before & after LSVT®.

[54] Liotti M, Ramig LO, Vogel D (2003). Hypophonia in Parkinson’s disease: neural correlates of voice treatment revealed by PET. *Neurology*.

[55] Narayana S, Fox PT, Zhang W (2010). Neural correlates of efficacy of voice therapy in Parkinson’s disease identified by performance-correlation analysis. *Human Brain Mapping*.

[56] Georgiou N, Iansek R, Bradshaw JL, Phillips JG, Mattingley JB, Bradshaw JA (1993). An evaluation of the role of internal cues in the pathogenesis of Parkinsonian hypokinesia. *Brain*.

[57] Bloem BR, Hausdorff JM, Visser JE, Giladi N (2004). Falls and freezing of Gait in Parkinson’s disease: a review of two interconnected, episodic phenomena. *Movement Disorders*.

[58] Behrman AL, Teitelbaum P, Cauraugh JH (1998). Verbal instructional sets to normalise the temporal and spatial gait variables in Parkinson’s disease. *Journal of Neurology Neurosurgery and Psychiatry*.

[59] Morris ME, Iansek R, Matyas TA, Summers JJ (1994). The pathogenesis of gait hypokinesia in Parkinson’s disease. *Brain*.

[60] Ebersbach G, Sojer M, Valldeoriola F (1999). Comparative analysis of gait in Parkinson’s disease, cerebellar ataxia and subcortical arteriosclerotic encephalopathy. *Brain*.

[61] Horak FB, Frank J, Nutt J (1996). Effects of dopamine on postural control in parkinsonian subjects: scaling, set, and tone. *Journal of Neurophysiology*.

[62] Berardelli A, Rothwell JC, Thompson PD, Hallett M (2001). Pathophysiology of bradykinesia in parkinson’s disease. *Brain*.

[63] Marchese R, Diverio M, Zucchi F, Lentino C, Abbruzzese G (2000). The role of sensory cues in the rehabilitation of Parkinsonian patients: a comparison of two physical therapy protocols. *Movement Disorders*.

[64] Nieuwboer A, Kwakkel G, Rochester L (2007). Cueing training in the home improves gait-related mobility in Parkinson’s disease: the RESCUE trial. *Journal of Neurology, Neurosurgery and Psychiatry*.

[65] Rubinstein TC, Giladi N, Hausdorff JM (2002). The power of cueing to circumvent dopamine deficits: a review of physical therapy treatment of gait disturbances in Parkinson’s disease. *Movement Disorders*.

[66] Dam M, Tonin P, Casson S (1996). Effects of conventional and sensory-enhanced physiotherapy on disability of Parkinson’s disease patients. *Advances in neurology*.

[67] McIntosh GC, Brown SH, Rice RR, Thaut MH (1997). Rhythmic auditory-motor facilitation of gait patterns in patients with Parkinson’s disease. *Journal of Neurology Neurosurgery and Psychiatry*.

[68] Morris ME, Iansek R, Matyas TA, Summers JJ (1996). Stride length regulation in Parkinson’s disease: normalization strategies and underlying mechanisms. *Brain*.

[69] Thaut MH, McIntosh GC, Rice RR, Miller RA, Rathbun J, Brault JM (1996). Rhythmic auditory stimulation in gait training for Parkinson’s disease patients. *Movement Disorders*.

[70] Müller V, Mohr B, Rosin R, Pulvermüller F, Müller F, Birbaumer N (1997). Short-term effects of behavioral treatment on movement initiation and postural control in Parkinson’s disease: a controlled clinical study. *Movement Disorders*.

[71] Miyai I, Fujimoto Y, Yamamoto H (2002). Long-term effect of body weight-supported treadmill training in Parkinson’s disease: a randomized controlled trial. *Archives of Physical Medicine and Rehabilitation*.

[72] Jobges EM, Spittler-Schneiders H, Renner CIE, Hummelsheim H (2007). Clinical relevance of rehabilitation programs for patients with idiopathic Parkinson syndrome. II: symptom-specific therapeutic approaches. *Parkinsonism and Related Disorders*.

[73] Lehman DA, Toole T, Lofald D, Hirsch MA (2005). Training with verbal instructional cues results in near-term improvement of gait in people with Parkinson disease. *Journal of Neurologic Physical Therapy*.

[74] Hirsch MA, Toole T, Maitland CG, Rider RA (2003). The effects of balance training and high-intensity resistance training on persons with idiopathic Parkinson’s disease. *Archives of Physical Medicine and Rehabilitation*.

[75] Dibble LE, Hale TF, Marcus RL, Droge J, Gerber JP, LaStayo PC (2006). High-intensity resistance training amplifies muscle hypertrophy and functional gains in persons with parkinson’s disease. *Movement Disorders*.

[76] Farley BG, Koshland GF (2005). Training BIG to move faster: the application of the speed-amplitude relation as a rehabilitation strategy for people with Parkinson’s disease. *Experimental Brain Research*.

[77] Schrag A, Dodel R, Spottke A, Bornschein B, Siebert U, Quinn NP (2007). Rate of clinical progression in Parkinson’s disease. A prospective study. *Movement Disorders*.

[78] Sage MD, Almeida QJ (2009). Symptom and gait changes after sensory attention focused exercise vs aerobic training in Parkinson’s disease. *Movement Disorders*.

[79] Mak MKY, Hui-Chan CWY (2008). Cued task-specific training is better than exercise in improving sit-to-stand in patients with Parkinson’s disease: a randomized controlled trial. *Movement Disorders*.

[80] Desmurget M, Grafton ST, Vindras P, Gréa H, Turner RS (2004). The basal ganglia network mediates the planning of movement amplitude. *European Journal of Neuroscience*.

[81] Duffy J (1995). *Motor Speech Disorders*.

[82] Rosenbek JC, LaPointe LL, Johns DF (1985). The dysarthrias: description, diagnosis, and treatment. *Clinical Management of Neurogenic Communicative Disorders*.

[83] Yorkston KM, Beukelman DR, Bell KR (1988). *Clinical Measurement of Dysarthric Speakers*.

[84] King LA, Horak FB (2009). Delaying mobility disability in people with parkinson disease using a sensorimotor agility exercise program. *Physical Therapy*.

[85] Rochester L, Hetherington V, Jones D (2005). The effect of external rhythmic cues (auditory and visual) on walking during a functional task in homes of people with Parkinson’s disease. *Archives of Physical Medicine and Rehabilitation*.

[86] Morris ME, Martin CL, Schenkman ML (2010). Striding out with Parkinson disease: evidence-based physical therapy for gait disorders. *Physical Therapy*.

[87] Kleim J, Jones T Principles of experience-dependent neural plasticity: implications for rehabilitation after brain damage.

[88] Demirci M, Grill S, McShane L, Hallett M (1997). A mismatch between kinesthetic and visual perception in Parkinson’s disease. *Annals of Neurology*.

[89] Jobst EE, Melnick ME, Byl NN, Dowling GA, Aminoff MJ (1997). Sensory perception in Parkinson disease. *Archives of Neurology*.

[90] Klockgether T, Borutta M, Rapp H, Spieker S, Dichgans J (1995). A defect of kinesthesia in Parkinson’s disease. *Movement Disorders*.

[91] Schneider JS, Diamond SG, Markham CH (1987). Parkinson’s disease: sensory and motor problems in arms and hands. *Neurology*.

[92] París AP, Saleta HG, de la Cruz Crespo Maraver M (2011). Blind randomized controlled study of the efficacy of cognitive training in Parkinson's disease. *Movement Disorders*.

[93] Brauer SG, Morris ME (2010). Can people with Parkinson’s disease improve dual tasking when walking?. *Gait and Posture*.

[94] Yogev-Seligmann G, Giladi N, Brozgol M, Hausdorff JM (2012). A training program to improve gait while dual tasking in patients with parkinson's disease: a pilot study. *Archives of Physical Medical Rehabilitation*.

[95] Countryman S, Ramig L (1993). Effects of intensive voice therapy on voice deficits associated with bilateral thalamotomy in Parkinson’s disease: a case study. *Journal of Medical Speech-Language Pathology*.

[96] Countryman S, Ramig L, Pawlas A (1994). Speech and voice deficits in Parkinsonian plus syndromes: can they be treated?. *Journal of Medical Speech-Language Pathology*.

[97] Spielman J, Mahler L, Halpern A, Gilley P, Klepitskaya O, Ramig L (2011). Intensive voice treatment (LSVTÒLOUD) for Parkinson's disease following deep brain stimulation of the subthalamic nucleus. *Journal of Communication Disorders*.

[98] Spielman J, Ramig LO, Will L, Halpern A, Petska J (2007). Effects of LSVT extended (LSVT-X) on voice and speech in Parkinson disease. *American Journal of Speech, Language Pathology*.

[99] Halpern A, Spielman J, Ramig L, Panzer I, Sharpley A, Gustafson H (2011). A novel way to measure speech intelligibility in individuals with Parkinson disease. *Movement Disorders*.

[100] Dumer AI, Borod JC, Oster H, Spielman JL, Rabin LA, Ramig LO (2011). Reduction of facial movement deficits in Parkinsons disease (PD) after Lee Silverman voice treatment (LSVT). Poster Presentations. *Movement Disorders*.

[101] Tripoliti E, Zrinzo L, Martinez-Torres I (2008). Effects of contact location and voltage amplitude on speech and movement in bilateral subthalamic nucleus deep brain stimulation. *Movement Disorders*.

[102] Taub E, Lum PS, Hardin P, Mark VW, Uswatte G (2005). AutoCITE: automated delivery of CI therapy with reduced effort by therapists. *Stroke*.

[103] Cherney LR, Halper AS (2008). Novel technology for treating individuals with aphasia and concomitant cognitive deficits. *Topics in Stroke Rehabilitation*.

[104] Manheim LM, Halper AS, Cherney L (2009). Patient-Reported Changes in Communication After Computer-Based Script Training for Aphasia. *Archives of Physical Medicine and Rehabilitation*.

[105] Tindall LR, Huebner RA, Stemple JC, Kleinert HL (2008). Videophone-delivered voice therapy: a comparative analysis of outcomes to traditional delivery for adults with Parkinson’s disease. *Telemedicine & E Health*.

[106] Constantinescu GA, Theodoros DG, Russell TG, Ward EC, Wilson SJ, Wootton R (2010). Home-based speech treatment for Parkinson’s disease delivered remotely: a case report. *Journal of Telemedicine and Telecare*.

[107] Theodoros DG, Constantinescu G, Russell TG, Ward EC, Wilson SJ, Wootton R (2006). Treating the speech disorder in Parkinson’s disease online. *Journal of Telemedicine and Telecare*.

[108] Theodoros D, Ramig L (2011). Telepractice Supported Delivery of LSVT® LOUD. *Perspectives on Neurophysiology and Neurogenic Speech and Language Disorders*.

[109] Halpern A, Matos C, Ramig L, Petska J, Spielman J, Will L (2005). Technology supported speech treatment for Parkinson’s disease. *Movement Disorders*.

[110] Hoffmann T, Russell T, Thompson L, Vincent A, Nelson M (2008). Using the Internet to assess activities of daily living and hand function in people with Parkinson’s disease. *NeuroRehabilitation*.

[111] Chumbler NR, Rose DK, Griffiths P (2010). Study protocol: home-based telehealth stroke care: a randomized trial for veterans. *Trials*.

[112] Ciucci MR, Ma ST, Fox C, Kane JR, Ramig LO, Schallert T (2007). Qualitative changes in ultrasonic vocalization in rats after unilateral dopamine depletion or haloperidol: a preliminary study. *Behavioural Brain Research*.

[113] Ciucci MR, Connor NP (2009). Dopaminergic influence on rat tongue function and limb movement initiation. *Experimental Brain Research*.

[114] Miller JE, Burkett ZD, Fox CM, White SA (2011). Vocal motor deficits in a songbird model of Parkinson's disease. Poster Presentations. *Movement Disorders*.

